# Oligoribonucleotide (ORN) Interference-PCR (ORNi-PCR): A Simple Method for Suppressing PCR Amplification of Specific DNA Sequences Using ORNs

**DOI:** 10.1371/journal.pone.0113345

**Published:** 2014-11-18

**Authors:** Naoki Tanigawa, Toshitsugu Fujita, Hodaka Fujii

**Affiliations:** Chromatin Biochemistry Research Group, Combined Program on Microbiology and Immunology, Research Institute for Microbial Diseases, Osaka University, Suita, Osaka, Japan; University of Houston, United States of America

## Abstract

Polymerase chain reaction (PCR) amplification of multiple templates using common primers is used in a wide variety of molecular biological techniques. However, abundant templates sometimes obscure the amplification of minor species containing the same primer sequences. To overcome this challenge, we used oligoribonucleotides (ORNs) to inhibit amplification of undesired template sequences without affecting amplification of control sequences lacking complementarity to the ORNs. ORNs were effective at very low concentrations, with IC_50_ values for ORN-mediated suppression on the order of 10 nM. DNA polymerases that retain 3′–5′ exonuclease activity, such as KOD and *Pfu* polymerases, but not those that retain 5′–3′ exonuclease activity, such as *Taq* polymerase, could be used for ORN-mediated suppression. ORN interference-PCR (ORNi-PCR) technology should be a useful tool for both molecular biology research and clinical diagnosis.

## Introduction

Polymerase chain reaction (PCR) is an essential method for molecular biological research. Amplification of multiple templates using common primers is widely used for cloning of members of gene families [1], [2] and detection of microorganisms in clinical diagnosis. In such applications, amplicons of abundant templates that contain the primer sequences can often predominate over those of less abundant species, making it difficult to detect amplification of minor species. Therefore, it is necessary to develop methods that efficiently suppress amplification of abundant templates but allow amplification of minor or desired templates containing the same primer sequences.

Several methods have been developed to achieve this goal. Blocking oligodeoxyribonucleotides (ODNs) or locked nucleic acids (LNAs) complementary to the specific sequences of known genes have been used to inhibit their amplification [3]–[7]. In these procedures, the 3′ termini of ODNs and LNAs must be modified so that they are not able to serve as primers for PCR reactions. The 3′ modifications used for this purpose include dideoxidization [4], nonbase-pairing tails [5], [6], and addition of a phosphate group [7]; however, these approaches are not feasible when used with DNA polymerases that have 3′–5′ exonuclease activity, because such enzymes can remove the 3′ modifications and thus allow the ODNs or LNAs to serve as primers. Therefore, these methods are applicable only when 3′–5′ exonuclease-deficient DNA polymerases such as Stoffel fragment or *Taq* polymerases are used [4]–[7]. Because 3′–5′ exonuclease activity is important for proofreading activity of DNA polymerases, the inability to use DNA polymerases that retain this activity may result in higher error rates in the amplification process.

Previous work showed that long RNAs (ca. 750 bases) could specifically inhibit amplification of targets by PCR [8]; however, this approach requires synthesis of RNA by *in vitro* transcription, which is expensive and time-consuming. Moreover, it remains unclear how tightly specificity of inhibition can be controlled using this strategy.

To overcome the aforementioned problems with conventional methods, we developed oligoribonucleotide (ORN) interference-PCR (ORNi-PCR) technology, in which ORNs are used to inhibit amplification of specific templates. This strategy has several advantages, namely, ORNs can be chemically synthesized easily and inexpensively, and because of their short length, it is straightforward to design ORNs for specific inhibition. Furthermore, ORNs can be easily removed by RNases for downstream applications and thus ORNi-PCR technology should be a useful tool for various applications in molecular biology research and clinical diagnosis.

## Results and Discussion

### Specific inhibition of PCR amplification using ORNs

**Figure 1:**
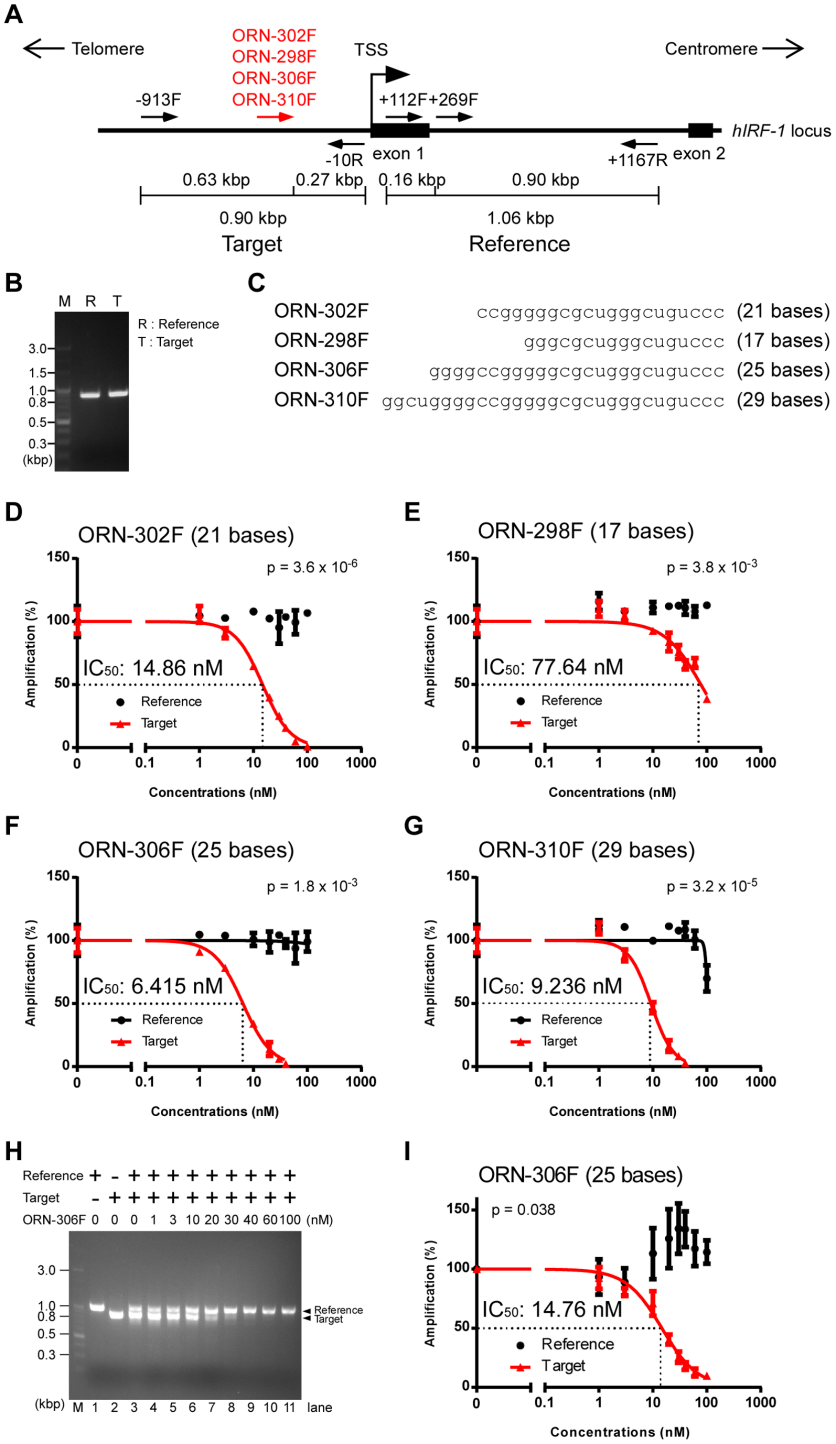
Inhibition of PCR amplification by ORNs of various lengths. (**A**) Positions of oligoribonucleotides (ORNs) in the target region. Primers were designed against the human *IRF-1* locus to amplify two neighboring regions. (**B**) Comparable amplification of the target and reference regions in the *IRF-1* locus. (**C**) Alignment of ORNs (5′–3′) of various lengths. (**D–G**) Dose responses of ORNs of various lengths. Real-time PCR amplification in the presence of various concentrations of ORNs was normalized against the value in the absence of ORNs. Dose-response curves were generated using the Prism software; Prism was not able to generate curves of the references for ORN-302F (**D**) and −298F (**E**). Data are represented as means ± S.D. (n = 3). (**H**) Suppression of amplification of the target template by ORN-306F in a reaction in which the target and reference primers were mixed. (**I**) Dose responses calculated for the condition used in (**H**). P-values for the differences in amplification between the reference and the target at an ORN concentration of 10 nM were calculated using Student’s t-test (**D–G, I**).

We reasoned that ORNs could suppress PCR amplification of specific sequences by annealing to the template, and sought to determine whether such ORN-mediated suppression occurs. As a model for testing, we designed primers complementary to the human *IRF-1* (*hIRF-1*) locus that amplify two neighboring regions 900 bp in size, namely, the reference region (hIRF1+269F and hIRF1+1167R) and the target region (hIRF1-913F and hIRF1-10R) (Figure 1A). The efficiency of amplification was comparable between the two regions (Figures 1B and S1). We then designed a 21-base ORN, ORN-302F, complementary to the target region (Figure 1A, C). As shown in Figure 1D, amplification of the target region was inhibited by increasing concentrations of ORN-302F, whereas amplification of the reference region was not inhibited. The IC_50_ of ORN-302F-mediated inhibition was 14.86 nM (Figure 1D). This result demonstrated the feasibility of ORN-mediated specific inhibition of PCR. In mechanistic terms, ORNs may hybridize to the target template and prevent DNA polymerases from extending the DNA strand from the primer, thereby suppressing amplification of the target template.

### Optimal lengths of ORNs for suppression of PCR

Next, we tested ORNs of various lengths (Figure 1C) and evaluated the efficiency with which they suppressed PCR. As shown in Figure 1E, the slightly shorter ORN-298F (17 bases) specifically suppressed amplification of the target; however, its IC_50_ value (77.64 nM) was markedly higher than that of the 21-base ORN-302F. By contrast, the longer ORN-306F (25 bases) also specifically suppressed amplification of the target, but with a slightly lower IC_50_ than that of ORN-302F (6.415 nM) (Figure 1F). An even longer ORN, the 29-base ORN-310F, had an IC_50_ (9.236 nM) comparable to that of ORN-306F, and also suppressed amplification of the reference template at a higher concentration (100 nM) (Figure 1G). These results indicated that ORNs with lengths of 21–25 bases can specifically inhibit amplification of target sequences with optimal IC_50_ values.

We validated the suppression of amplification of the target template by ORN-306F in a reaction in which the target and reference primers were mixed (Figure 1H and I). To distinguish the bands of the reference (1.06 kbp) and target (0.90 kbp) amplicons by agarose gel electrophoresis, we used the hIRF1+112F primer instead of hIRF1+296F (Figure 1A). Suppression efficiency was calculated semi-quantitatively by image-processing analysis. The calculated IC_50_ from this experiment (14.76 nM) (Figure 1I) was comparable to that determined by real-time PCR (6.415 nM) (Figure 1F).

### Selection of ORNs for effective suppression

**Figure 2:**
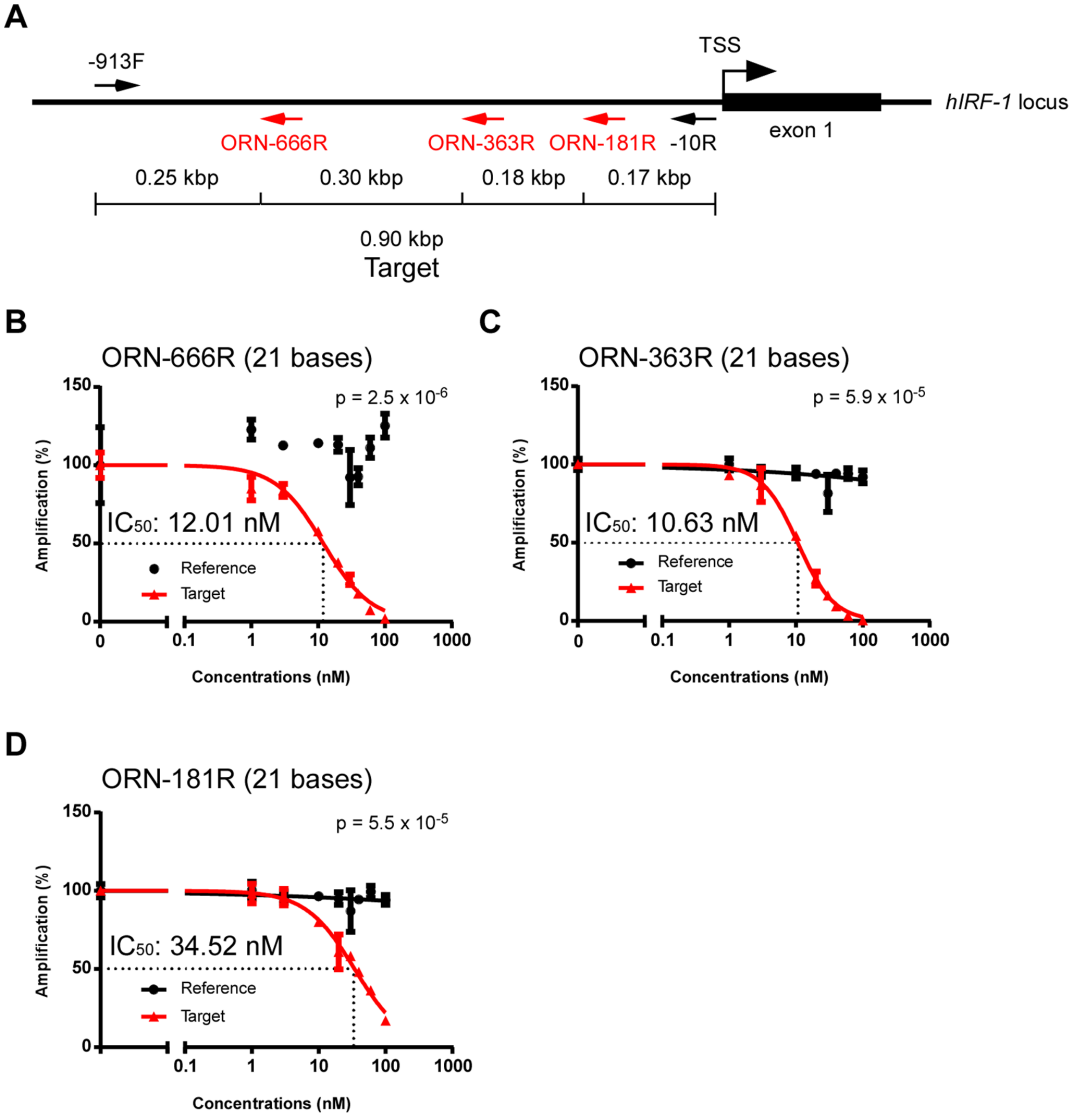
Effects of ORN position on inhibition of PCR amplification. (**A**) Positions of ORNs in the target region. (**B–D**) Dose responses of ORNs. Real-time PCR amplification in the presence of various concentrations of ORNs was normalized against the value in the absence of ORNs. Dose-response curves were generated using the Prism software; Prism was not able to generate a curve of the reference for ORN-666R (**B**). Data are represented as means ± S.D. (n = 3). P-values for the differences in amplification between the reference and the target at an ORN concentration of 10 nM were calculated using Student’s t-test (**B–D**).

Next, we examined how easily optimal ORNs can be found in the target regions by designing 21-base ORNs to hybridize to various positions within the target sequence (ORN-666R, -363R and -181R) (Figure 2A). All of them efficiently suppressed amplification of the target sequence, although the IC_50_ of ORN-181R (34.52 nM) was higher than those of the other two (12.01 and 10.63 nM, respectively) (Figure 2B–D). These results suggested that ORNs can be designed flexibly in target regions, and that optimal ORNs can be easily found empirically.

### Potential synergistic or additive effects of multiple ORNs

**Figure 3:**
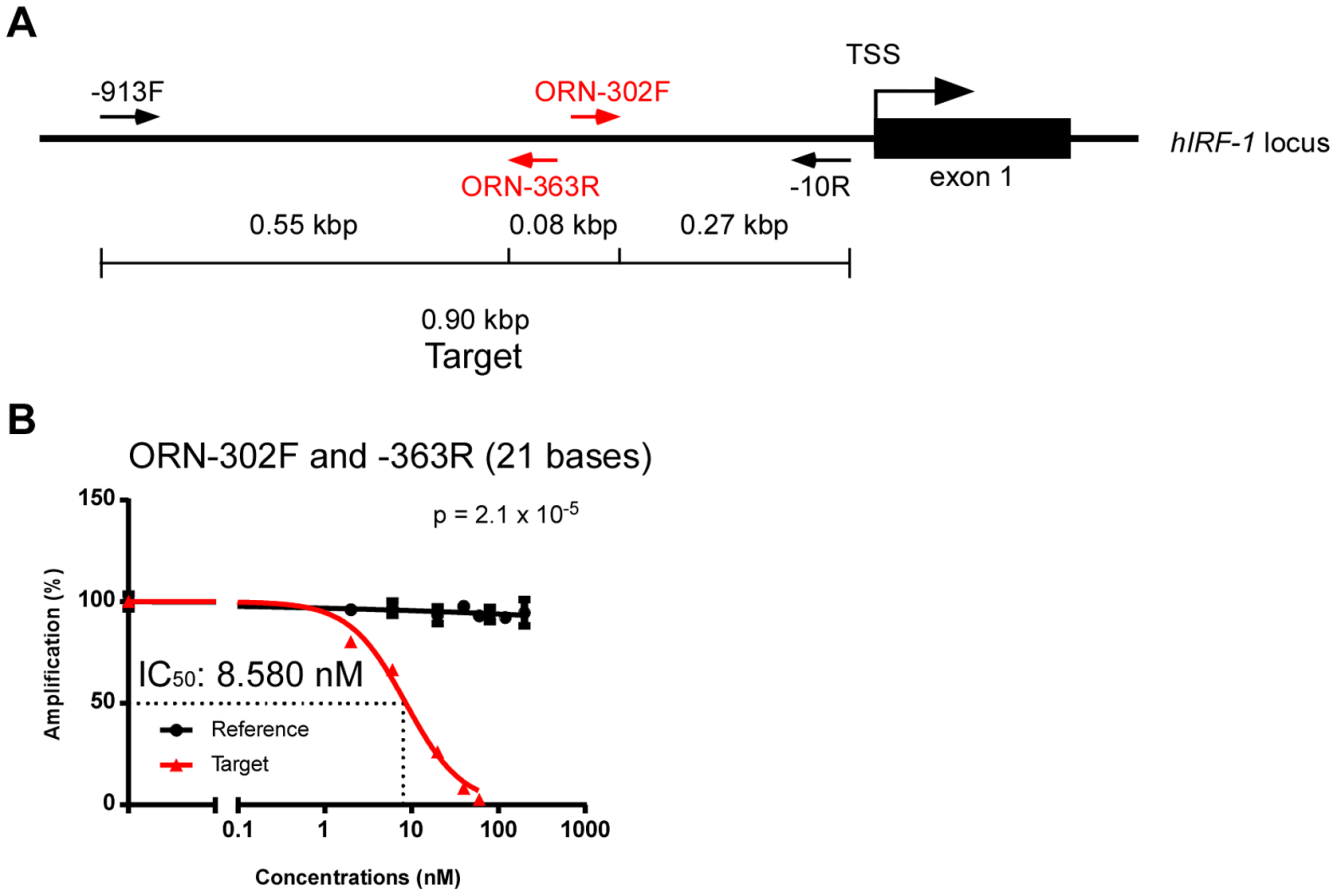
Inhibitory effects of multiple ORNs on PCR amplification. (**A**) Positions of ORNs in the target region. (**B**) Dose responses of multiple ORNs (ORN-302F and -363R). Real-time PCR amplification in different concentrations of ORNs was normalized against the value in the absence of ORNs. Dose-response curves were generated using the Prism software. Data are represented as means ± S.D. (n = 3). P-values for the differences in amplification between the reference and the target at a total ORN concentration of 20 nM (i.e., 10 nM for each ORN) were calculated using Student’s t-test.

Next, we investigated whether the use of multiple ORNs had a synergistic or additive effect on suppression of amplification. When a combination of ORN-302F and -363R was used, which are oriented in opposite directions (Figure 3A), the IC_50_ was 8.580 nM (Figure 3B). This value was slightly lower than that of ORN-302F (Figure 1D) or -363R (Figure 2C) used separately, but did not represent a significant improvement. This result suggested that, in practice, a single ORN would be sufficient for near-optimal suppression of PCR amplification.

### Specific inhibition of target sequences by ORNi-PCR in competitive settings

**Figure 4:**
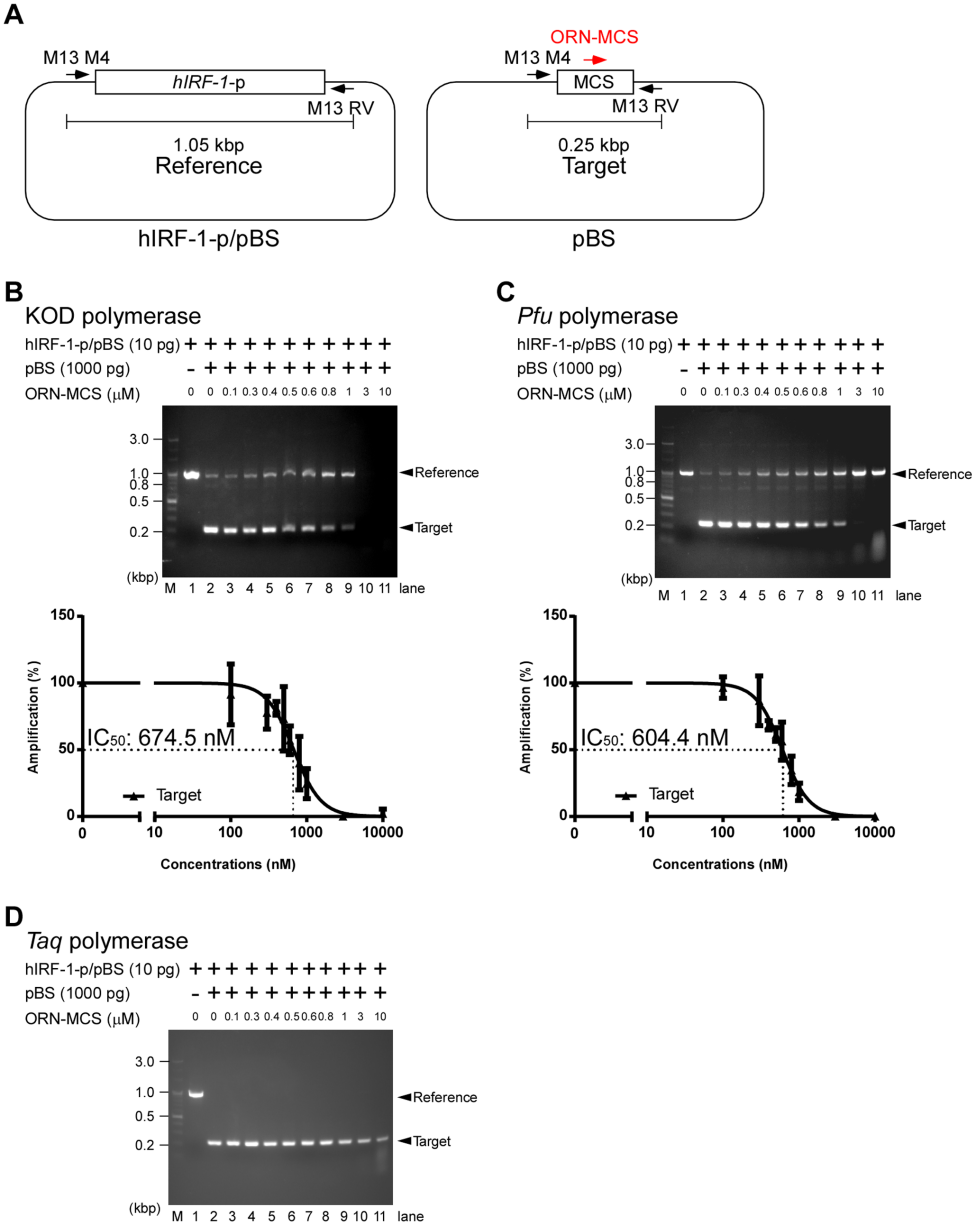
ORN-mediated inhibition of PCR amplification in a competitive setting. (**A**) Scheme for ORNi-PCR in a competitive setting. ORNi-PCR can be used for specific inhibition of target sequences while allowing amplification of reference sequences containing the same primer sequences. We designed an ORN targeting the multiple cloning site (MCS) of pBluescript-SK+ (pBS) (ORN-MCS). PCR amplification of the target and reference sequence in the presence or absence of different concentrations of ORNs, using KOD polymerase (**B**), *Pfu* polymerase (**C**), or *Taq* polymerase (**D**). Gel images (upper panels) are representative of three independent experiments. The lower panels show dose-response curves of ORN-mediated inhibition.

Next, we investigated whether ORNi-PCR can be used for specific inhibition of target sequences while allowing amplification of non-target templates containing the same primer sequences. In these experiments, we used the template plasmids pBluescript-SK+ (pBS) and a pBS-derived plasmid containing *hIRF-1* promoter sequence (hIRF-1-p/pBS) (Figure 4A). Because the sequence of *hIRF-1* promoter was inserted into *Kpn* I and *Sac* I sites of pBS, hIRF-1-p/pBS lacks the multiple cloning site (MCS) of pBS. hIRF-1-p/pBS served as an efficient template for PCR amplification using the M13 primers with the KOD DNA polymerase (Figure 4B, upper panel, lane 1). When pBS and hIRF-1-p/pBS were mixed in a 100∶1 ratio, PCR reactions resulted in selective amplification of the sequence derived from pBS (Target) but poor amplification of the sequence from hIRF-1-p/pBS (Reference) (Figure 4B, upper panel, lane 2). This selective amplification might have been caused by the lower concentration of hIRF-1-p/pBS, as well as the longer insert size of the *hIRF-1*-p sequence. Therefore, we designed an ORN targeting the multiple cloning site of pBS (ORN-MCS) (Figure 4A) and tested whether it could inhibit amplification of the pBS sequence. As shown in Figure 4B (upper panel, lane 9), ORN-MCS effectively inhibited amplification of the target sequence at a concentration of 1 µM, and amplification of the reference sequence in the same reaction increased. At higher concentrations (3 and 10 µM), ORN-MCS non-specifically suppressed amplification of the reference sequence, as well as the target sequence, when KOD polymerase was used (Figure 4B, upper panel, lanes 10 and 11). We speculate that KOD polymerase might be non-specifically inhibited by the ORN through direct binding to the active site. These results suggested that ORNi-PCR can be used for selective amplification of minor templates that are otherwise more difficult to amplify.

Next, we examined whether other DNA-dependent DNA polymerases can be used for ORNi-PCR. PCR reactions using the *Pfu* DNA polymerase in the same setting described above suppressed amplification of the target sequence, but augmented amplification of the *hIRF-1* promoter region in hIRF-1-p/pBS (Figure 4C, upper panel). The calculated IC_50_ (604.4 nM) was comparable to that obtained using KOD polymerase (674.5 nM) (Figure 4C, lower panel). The IC_50_ values in these experimental settings, using plasmid DNAs as templates (Figure 4B and C), were much higher than those for ORNi-PCR using genomic DNA as the template (6–78 nM) (Figures 1–3). Because plasmid DNA is of much lower complexity than genomic DNA, PCR amplification of plasmid DNA is much more efficient; consequently, higher concentrations of ORNs may be required for effective suppression.

On the other hand, in PCR reactions using *Taq* DNA polymerase, increasing concentrations of ORN-MCS (up to 10 µM) gradually suppressed amplification of the target sequence, but the suppression was never complete (Figure 4D). In contrast to PCR reactions using the KOD and *Pfu* polymerases, we failed to detect amplification of the reference sequence when *Taq* was used; thus these results showed that KOD and *Pfu* are suitable for ORNi-PCR, whereas *Taq* is not. There are several salient differences between these enzymes, namely, the KOD and *Pfu* DNA polymerases are α-type DNA polymerases that retain 3′–5′ exonuclease activity but not 5′–3′ exonuclease activity [9]–[11], whereas *Taq* DNA polymerase belongs to the Pol I–type DNA polymerase family and retains 5′–3′ exonuclease activity but not 3′–5′ exonuclease activity [12]. Our results suggested that α-type DNA polymerases, but not Pol I-type DNA polymerases, might be compatible with ORNi-PCR. It is possible that 5′–3′ exonuclease activity may remove ORNs bound to the target sequence, as DNA polymerase I does in prokaryotic DNA replication [13]. The fact that α-type DNA polymerases can be used for ORNi-PCR is advantageous, because 3′–5′ exonuclease activity is important for proofreading [14]. In this regard, other methods such as blocking ODNs or LNAs are not compatible with α-type DNA polymerases, because 3′ modifications of blocking ODNs or LNAs may be removed by 3′–5′ exonuclease activity [3]. Therefore, ORNi-PCR might enable more accurate amplification while effectively suppressing target amplification.

**Figure 5:**
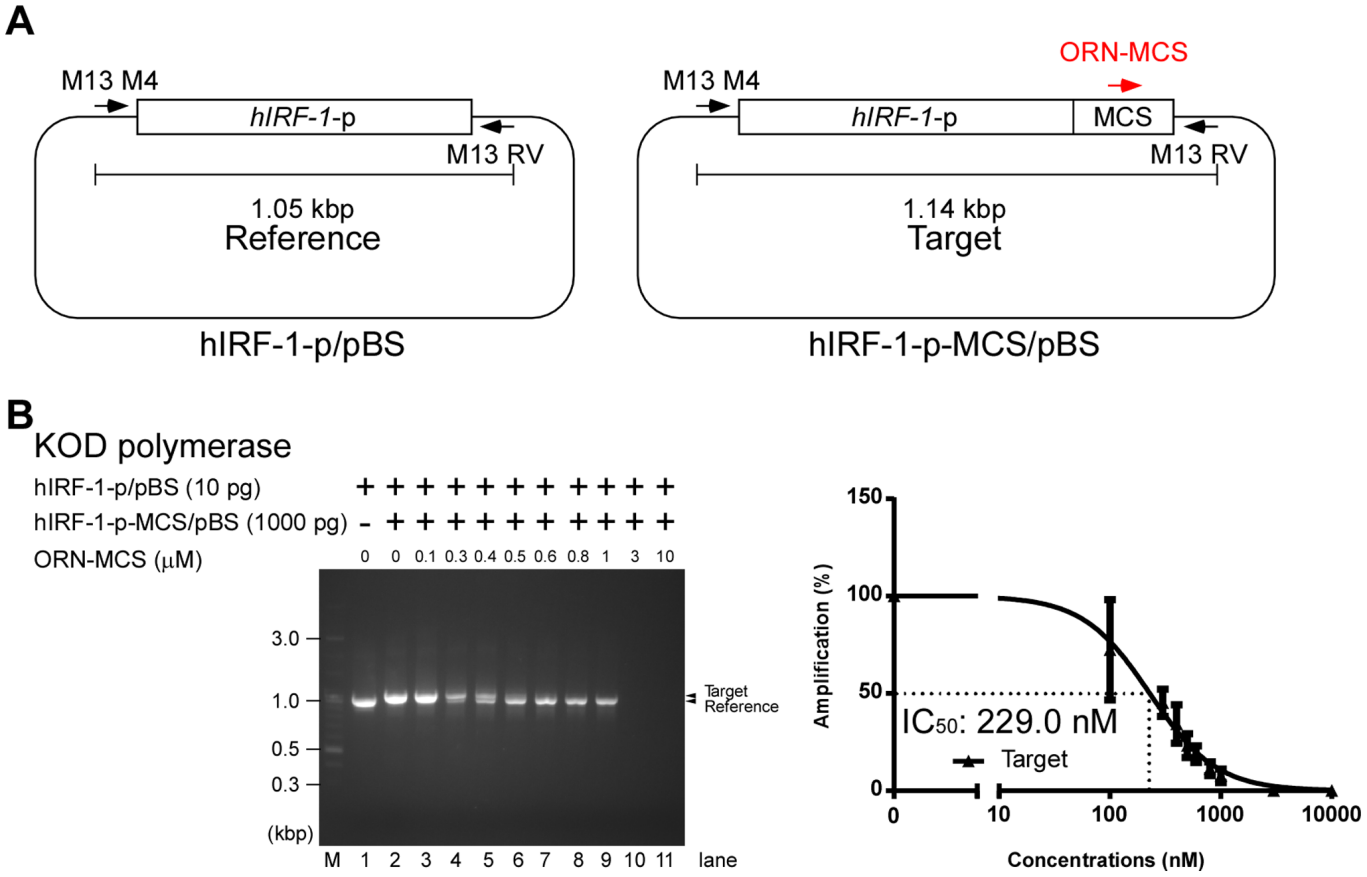
ORN-mediated inhibition of PCR amplification of a target sequence similar to the reference sequence, in a competitive setting. (**A**) Scheme for ORNi-PCR using a target sequence similar to the reference sequence in a competitive setting. (**B**) PCR amplification of the target sequence and reference sequence in the absence or presence of different concentrations of ORNs using the KOD polymerase. The gel image (left panel) is representative of three independent experiments. The right panel shows a dose-response curve of ORN-mediated inhibition.

Next, we investigated whether ORN-MCS could specifically inhibit a target sequence similar to the reference sequence. To this end, we constructed hIRF-1-p-MCS/pBS (Target), which is distinguished from hIRF-1-p/pBS (Reference) by the presence of the pBS MCS (Figure 5A). When hIRF-1-p-MCS/pBS and hIRF-1-p/pBS were mixed in a 100∶1 ratio, PCR reactions selectively amplified the reference sequence (Figure 5B, left panel, lane 2). ORN-MCS inhibited amplification of the target sequence, whereas amplification of the reference sequence was elevated in the same reaction mixture (Figure 5B, left panel, lanes 4–9). The calculated IC_50_ was 229.0 nM (Figure 5B, right panel). As shown in Figure 4B, ORN-MCS non-specifically suppressed amplification of the reference sequence at higher concentrations (Figure 5B, left panel, lanes 10 and 11). This result suggested that ORNi-PCR can be used for selective amplification of minor templates that are similar to abundant templates.

**Figure 6:**
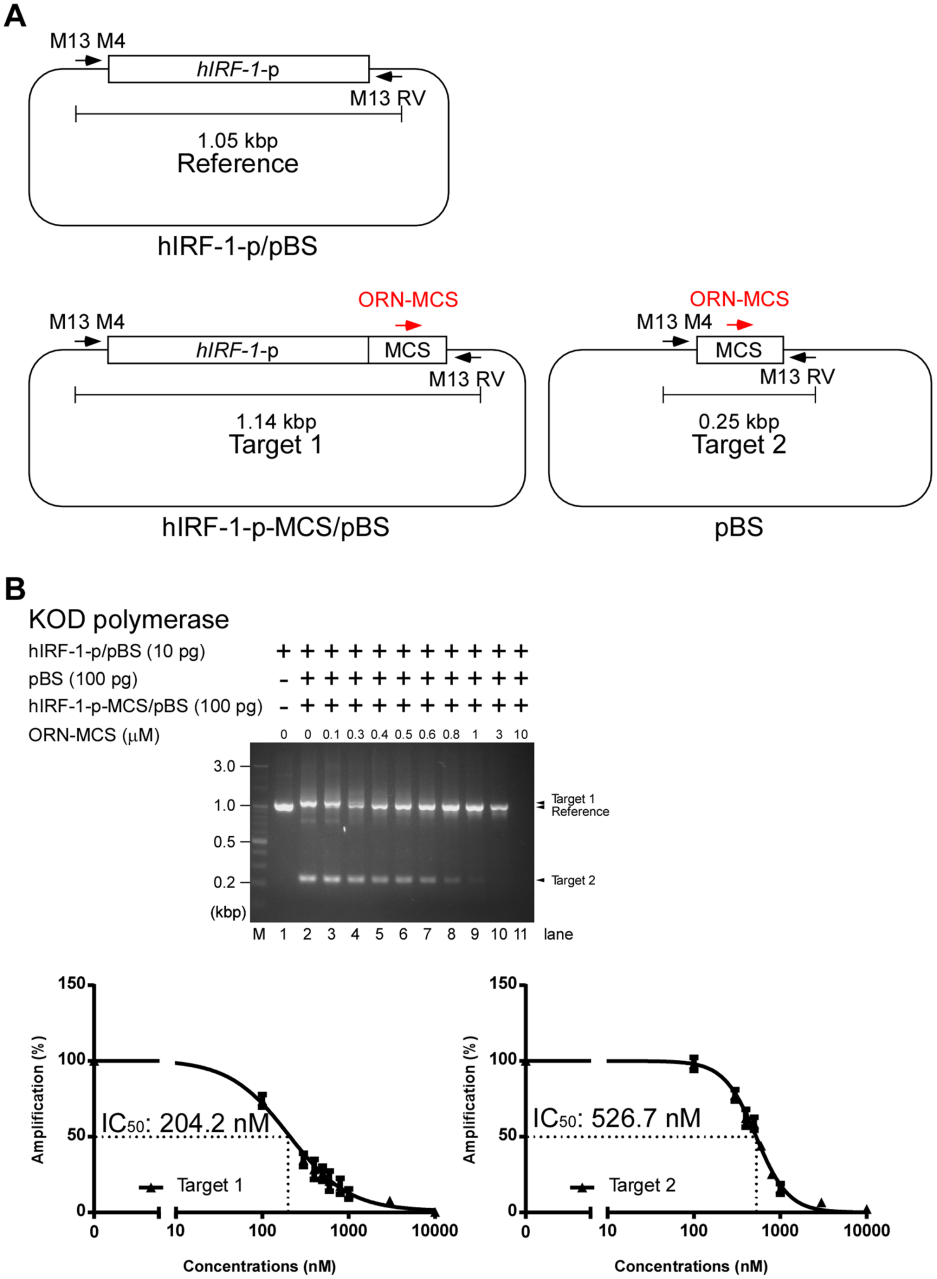
ORN-mediated inhibition of PCR amplification of multiple target sequences in a competitive setting. (**A**) Scheme for ORNi-PCR using multiple target sequences in a competitive setting. (**B**) PCR amplification of target sequences and the reference sequence in the presence or absence of various concentrations of ORNs, using KOD polymerase. The gel image (upper panel) is representative of three independent experiments. The lower right and left panels show dose-response curves of ORN-mediated inhibition for Targets 1 and 2, respectively.

Finally, we investigated whether ORN-MCS can specifically inhibit multiple target sequences. In these experiments, we used the template plasmids pBS and hIRF-1-p-MCS/pBS and the reference plasmid hIRF-1-p/pBS (Figure 6A). When hIRF-1-p-MCS/pBS (Target 1), pBS (Target 2), and hIRF-1-p/pBS (Reference) were mixed in a 10∶10∶1 ratio, PCR reactions selectively amplified Targets 1 and 2 but only marginally amplified the Reference (Figure 6B, upper panel, lane 2). ORN-MCS could inhibit amplification of both target sequences (Figure 6B, upper panel, lanes 4–10). By contrast, amplification of the Reference was elevated in the same reaction mixture (Figure 6B, upper panel, lanes 4–10). The calculated IC_50_ values were 204.2 nM and 526.7 nM for Targets 1 and 2, respectively (Figure 6B, lower panels). This result suggested that ORNi-PCR can be used for selective amplification of minor templates in the presence of multiple abundant templates.

## Conclusions

In this study, we demonstrated that ORNs specifically inhibit PCR amplification of target templates containing sequences complementary to the ORNs. For optimal suppression, ORNs should be around 21–25 bases long. α-type DNA-dependent polymerases retaining 3′–5′ exonuclease activity, such as the KOD and *Pfu* polymerases, can be used for ORNi-PCR. Regarding the mechanism, ORNs may hybridize to the target template and prevent DNA polymerases from extending the DNA strand from the primer, thereby suppressing amplification of the target template. ORNi-PCR technology will be a useful tool in both molecular biology research and clinical diagnosis. In addition, the ORN-mediated suppression strategy might also be applied to other types of template-dependent synthesis of nucleic acids, e.g., DNA-dependent DNA synthesis, transcription, and reverse transcription.

## Materials and Methods

### Oligonucleotides

Primers and ORNs used in this study were chemically synthesized (Greiner), and their sequences are provided in Table 1, Figure 1C, and Table S1.

**Table 1:** ORNs used in this study and their efficiency of suppression in ORNi-PCR.

Number	Name	ORN sequence (5′–3′)	Length(base)	IC_50_ (nM)	Experiments
R4	ORN-302F	ccgggggcgcugggcuguccc	21	14.86	Figures 1D and 3B
R6	ORN-298F	gggcgcugggcuguccc	17	77.64	Figure 1E
R5	ORN-306F	ggggccgggggcgcugggcuguccc	25	6.415	Figure 1F
				14.76	Figure 1I
R7	ORN-310F	ggcuggggccgggggcgcugggcuguccc	29	9.236	Figure 1G
R10	ORN-666R	ggccgcugcuggcacagcccc	21	12.01	Figure 2B
R9	ORN-363R	cacccuccuggcggggcgggg	21	10.63	Figures 2C and 3B
R8	ORN-181R	cacccucuccggccgggcgcc	21	34.52	Figure 2D
R11	ORN-MCS	agagcggccgccaccgcggug	21	674.5	Figure 4B
				604.4	Figure 4C
				229.0	Figure 5B
				204.2	Figure 6B
				526.7	Figure 6B

Genomic DNA was purified from the 293T cell line [15], [16]. For analysis of the amplification efficiencies of target and reference templates, PCR reactions were performed in mixtures containing 40 ng of 293T genomic DNA, 1× Buffer provided with KOD FX, 0.32 mM dNTPs, 0.25 µM of each primer, and 0.2 µL of KOD FX (Toyobo) in a 10 µL volume. The reactions were carried out with an initial denaturation at 94°C for 2 min, followed by 28 cycles of denaturation at 98°C for 10 sec, primer annealing and extension at 68°C for 1 min, and final extension at 68°C for 2 min. Real-time PCR reaction mixtures contained 40 ng of 293T genomic DNA, 0.2 µM of each primer, different concentrations of ORN, 1× ROX reference dye, and 5 µL of KOD SYBR qPCR Mix (Toyobo) in 10 µL reactions. The reactions were carried out with an initial denaturation at 98°C for 2 min, followed by 40 cycles of denaturation at 98°C for 10 sec, primer annealing at 60°C for 10 sec, and extension at 68°C for 1 min. The specificity of PCR products was confirmed by performing a dissociation curve analysis at 95°C for 15 sec, 60°C for 15 sec, and at 99°C for 15 sec. PCR amplification was quantitated on a 7900HT Fast Real-Time PCR System (Applied Biosystems). PCR products were subsequently electrophoresed on 2% agarose gels to confirm the presence of a unique amplicon of the expected size. All samples were amplified in triplicate, and IC_50_ values were calculated from the means using the Prism software (GraphPad).

For endpoint analysis, PCR reactions were performed with 40 ng of 293T genomic DNA, 1× Buffer provided with KOD -Plus- Ver.2, 0.2 mM dNTPs, 1.5 mM MgSO_4_, 0.3 µM of each primer, and 0.2 µL of KOD -Plus- Ver.2 (Toyobo) in a 10 µL volume. The reactions were carried out with an initial denaturation at 94°C for 2 min, followed by 29 cycles of denaturation at 98°C for 10 sec, primer annealing at 60°C for 30 sec, and extension at 68°C for 1 min. The PCR products were subsequently electrophoresed on 2% agarose gels.

### Statistical analysis

P-values were calculated using the Excel software (Microsoft) using Student’s t-test.

### Densitometric analysis

Densitometric analyses were performed using the ImageJ software (National Institutes of Health), and IC_50_ values were calculated from the means using the Prism software.

### Plasmid construction

To construct hIRF-1-p/pBS, the sequence of the *hIRF-1* promoter was amplified using the hIRF1-913F_KpnI and hIRF1-10R_SacI primers with 293T genomic DNA as the template. The resulting amplicon was cleaved with *Kpn*I and *Sac*I, and ligated into pBluescript-SK+ (Stratagene) digested with the same enzymes.

To generate hIRF-1-p-MCS/pBS, the sequence of the *hIRF-1* promoter was amplified using the hIRF1-913F_KpnI and hIRF1-10R_XhoI primers with 293T genomic DNA as template. The resulting amplicon was cleaved with *Kpn*I and *Xho*I, and ligated into pBluescript-SK+ (Stratagene) digested with the same enzymes.

### ORNi-PCR using plasmid DNA as templates

PCR was performed using KOD -Plus- Ver.2 (Toyobo) or *TaKaRa Taq* (Takara) in the presence of 1× Buffer provided with enzymes, 0.2 mM dNTPs, 0.3 µM each of M13 Primer M4 and M13 Primer RV, and various concentrations of ORN-MCS. When KOD was used, 1.5 mM MgSO_4_ was added. Templates were mixtures of 100 or 1,000 pg of the target plasmids (pBS and/or hIRF-1-p-MCS/pBS) and 10 pg of the reference plasmid (hIRF-1-p/pBS). The reactions were carried out with an initial denaturation at 94°C for 2 min, followed by 30 cycles of denaturation at 98°C for 10 sec, primer annealing at 55°C for 30 sec, and extension at 68°C for 1 min. When *TaKaRa Taq* was used, an additional step of 68°C for 2 min was added at the end of the reaction.

PCR was also performed with Pfu-X (Greiner) in the presence of 1× Buffer provided with Pfu-X, 0.2 mM dNTPs, 5% DMSO, 0.4 µM each of M13 Primer M4 and M13 Primer RV, and various concentrations of ORN-MCS. The reactions were carried out with an initial denaturation at 95°C for 2 min, followed by 30 cycles of denaturation at 95°C for 20 sec, primer annealing at 55°C for 20 sec, extension at 68°C for 30 sec, and a final extension at 68°C for 30 sec.

## Supporting Information

Figure S1 Comparable amplification of target and reference regions in the *IRF-1* locus by real-time PCR.
(PDF)

Table S1 PCR primers used in this study.
(PDF)
