# Supplementary material for: Oligoribonucleotide (ORN) Interference-PCR (ORNi-PCR): A Simple Method for Suppressing PCR Amplification of Specific DNA Sequences Using ORNs
Source: PLoS One. 2014 Nov 18;9(11):e113345. doi: 10.1371/journal.pone.0113345 (PMC4236179; doi:10.1371/journal.pone.0113345)
Supplement: Figure S1 — Comparable amplification of target and reference regions in the IRF-1 locus by real-time PCR. (PDF) [file pone.0113345.s001.pdf]

Figure S1

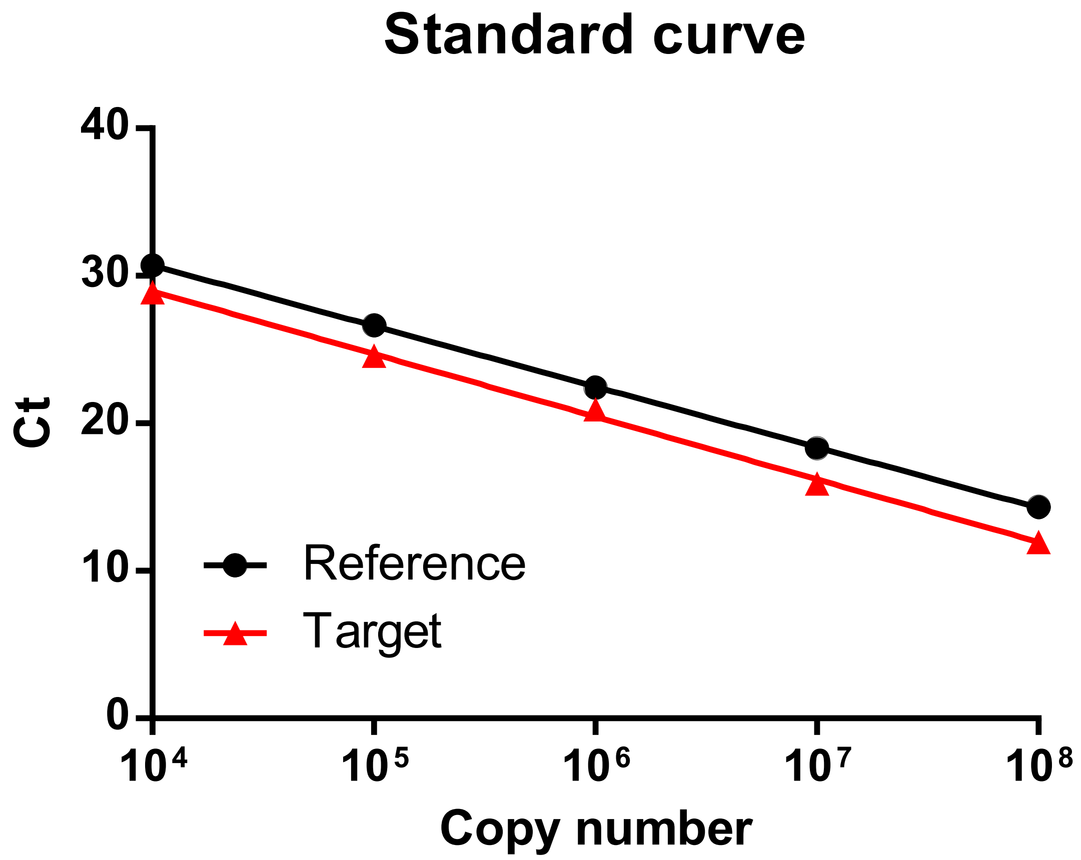

Standard curve
40
30
20
10
0
Ct
Reference
Target
10⁴
10⁵
10⁶
10⁷
10⁸
Copy number

Supplemental Figure Legends

Figure S1. Comparable amplification of target and reference regions in the *IRF-1* locus. For comparison of the amplification efficiencies of target and reference templates, real-time PCRs were performed with mixtures containing various concentrations of PCR products (from Figure 1B) as templates, 0.2 M of each primer, 1× ROX reference dye, and 5 µL of KOD SYBR qPCR Mix (Toyobo) in a 10 µL volume. The reactions were carried out with an initial denaturation at 98°C for 2 min, followed by 40 cycles of denaturation at 98°C for 10 sec, primer annealing at 60°C for 10 sec, and extension at 68°C for 1 min. PCR amplification was quantitated on a 7900HT Fast Real-Time PCR System (Applied Biosystems). All samples were amplified in duplicate, and mean values were used to draw a standard curve. The efficiency of amplification was comparable between the reference and the target regions.
