# Supplementary material for: Oligoribonucleotide (ORN) Interference-PCR (ORNi-PCR): A Simple Method for Suppressing PCR Amplification of Specific DNA Sequences Using ORNs
Source: PLoS One. 2014 Nov 18;9(11):e113345. doi: 10.1371/journal.pone.0113345 (PMC4236179; doi:10.1371/journal.pone.0113345)
Supplement: Table S1 — PCR primers used in this study. (PDF) [file pone.0113345.s002.pdf]

Table S1. PCR primers used in this study

Number	Name	Sequence (5'-3')	Experiments
27242	hIRF1-913F	ctctgttccctaaagccagccagggggagt	ORNi-PCR in Figures 1-3
27241	hIRF1-10R	gcggcgccaccgagcaatccaaacacttag	ORNi-PCR in Figures 1-3
27645	hIRF1+112F	ccggcaacctctgccttcttccctcttcca	ORNi-PCR in Figures 1H, I
27377	hIRF1+269F	aataaaaggcgcgcggggcaccaggaagtg	ORNi-PCR in Figures 1B-G, 2, 3
27378	hIRF1+1167R	tcagtgcggaggggagctgcgctggaataa	ORNi-PCR in Figures 1-3
27456	hIRF1-913F_KpnI	ctggtaccctctgttccctaaagccagccagggggagt	Construction of hIRF-1-p/pBS and hIRF-1-p-MCS/pBS
27457	hIRF1-10R_SacI	cgagagctcgcggcgccaccgagcaatccaaacacttag	Construction of hIRF-1-p/pBS
27646	hIRF-1-10R_XhoI	tgccctcgaggcggcgccaccgagcaatccaaacacttag	Construction of hIRF-1-p-MCS/pBS
26446	M13 Primer M4	gttttcccagtcacgac	ORNi-PCR in Figures 4-6
26445	M13 Primer RV	gtcatagctgtttcctg	ORNi-PCR in Figures 4-6
